# A Cross-Sectional Comparative Study of the Performance of the Widal Test and the Typhidot Immunoassay for Typhoid Fever Diagnosis in the West Region of Cameroon

**DOI:** 10.1155/2021/8279122

**Published:** 2021-08-07

**Authors:** Karimo Ousenu, Innocent Mbulli Ali, Leonard Fonkeng Sama, Marcel Nsangou Ndam, Thibau Florant Tchouangueu, Christopher Bonglavnyuy Tume

**Affiliations:** ^1^Research Unit of Microbiology and Antimicrobial Substances, Department of Biochemistry, Faculty of Science, University of Dschang, Dschang, Cameroon; ^2^MARCAD Program, The Biotechnology Centre, University of Yaoundé 1, Yaoundé, Cameroon; ^3^Higher Institute of Applied Science, Gulf of Guinea University Institute, Douala, Cameroon; ^4^Dschang District Hospital, Dschang Health District, Dschang, West Region, Cameroon; ^5^Department of Biochemistry, Faculty of Science, University of Bamenda, Bamenda, Cameroon

## Abstract

**Background:**

The diagnosis of typhoid fever based on the Widal slide agglutination test remains a major hurdle in developing countries due to varied perceptions of the value of the Widal test in determining clinical decision-making. We undertook a study to evaluate the diagnostic performance of the Widal test and the Typhidot immunoassay in patients suspected of having typhoid fever in the Menoua division, West Region of Cameroon.

**Methods:**

Blood and stool samples were collected from 558 consenting febrile patients on the basis of suspicion of typhoid fever. These patients attended three district health services of the Menoua division between April 2018 and September 2019. These patients had clinical symptoms suggestive of typhoid fever as determined by their consultant. Serum was used for the Widal slide agglutination test and for the Typhidot rapid immunoassay test based on manufacturer's guidelines. A composite reference of fever plus positive coproculture for *Salmonella typhi* and *Salmonella paratyphi* was used as the reference. The sensitivity, specificity, and predictive values of the positive and negative tests were calculated as well as Cohen's kappa for agreement between the two tests.

**Results:**

Of 558 patients, 12.90% tested positive for the reference method, 57.17% tested positive for the Widal slide agglutination test, while 15.59% were positive for Typhidot-IgM. The overall sensitivity, specificity, and predictive values of the positive and negative tests were 80.56%, 94.03%, 66.6%, and 97.03% for Typhidot-IgM and 94.44%, 48.35%, 21.32%, and 98.33% for the Widal slide agglutination test, respectively. Cohen's kappa estimates were 0.1660 (0.121–0.211) and 0.386 (0.312–0.460) for the Widal test and Typhidot immunoassay for 53.6% and 76.16% agreements of all observations, respectively.

**Conclusion:**

The Widal test was found to have a lower predictive value for the diagnosis of typhoid fever in our setting. However, the Typhidot test, although better, was not ideal. Diagnosis of typhoid fever should therefore rely on adequate clinical suspicion and a positive Typhidot test to improve the clinical management of typhoid fever in our setting.

## 1. Background

Enteric fever caused by *Salmonella typhi* and *Salmonella paratyphi* remains a major burden in developing countries due to varied perceptions of the value of the Widal test in determining clinical decision-making. The burden of typhoid fever, worldwide, shows that it causes 16.6 million new infections and about 600,000 deaths each year [1]. However, the incidence of typhoid fever has dropped to about 10/100,000 population/year in developed countries due to improved living standard, proper hygiene and sanitation, and better healthcare systems, but the incidence is still higher, 100/100,000 population/year, in less developed countries [2]. A key challenge to the effective control of typhoid fever is related to poor diagnosis. Diagnosis of typhoid fever in clinical settings is complicated because of overlapping symptoms with other common infections such as malaria, dengue, and viral enteritis [3–5]. For proper diagnosis, a test with a good diagnostic performance, especially in children with febrile diseases [6, 7], is of great importance. In addition, the misuse of antibiotics via automedication makes diagnosis difficult on a clinical basis [8]. The gold standard for the diagnosis of enteric fever is blood culture, but this test not only has a poor sensitivity in clinical settings but also is time consuming and expensive for patients and clinics in remote settings where culture facilities may not be always available and the population is poor [9]. The main diagnostic in such settings is based on the Widal slide agglutination test which is difficult to interpret for several reasons: cross-reactivities, time lag between infection and production of antibodies, and persistence of target antibodies long after treatment with very low correlation with active disease [10, 11]. The tube dilution technique enables a quantification of specific antibodies, and a change in titer can indicate active disease, but it is not very accessible. Many studies suggest that the Typhidot test, a plausible alternative based on the detection of antibody production against the outer membrane preparation common to *Salmonella typhi* and *Salmonella paratyphi*, has better performance characteristics. However, variations in sensitivity and specificity in the diagnosis of enteric fever among adults and children have also been noted [12–14]. The assay gives good results during early infections with a sensitivity of 68–95% and a specificity of 75–95% [15, 16]. An increase in the negative predictive value is important in endemic areas [17]. Recommendations from the World Health Organization for typhoid rapid antibody testing [18] and some studies that have evaluated the Typhidot ability to detect antibodies have shown variations in sensitivity and specificity [15] in different settings. Although comparative studies have relied on the use of imperfect gold standard tests, a composite reference has been suggested to improve diagnostic values, but no agreement has been reached on which combinations can form a good composite reference standard, Storey et al. [19]. In this study, we undertook to evaluate the diagnostic performance of the Widal and Typhidot tests against a composite reference made of a combination of fever (≥37.50 C), 3–7 days or more, and a positive stool culture test for *Salmonella typhi* and/or *paratyphi* in combination with one or more of the following clinical symptoms: persistent headache, abdominal discomfort, vomiting, and nausea. The choice of this combination was based on the feasibility and independence of the tests conditional to the disease, and although less specific, measures were taken to exclude other common febrile conditions such as malaria and respiratory tract infections. This was done in a bid to find a local strategy to better diagnose typhoid fever.

## 2. Methods

### 2.1. Study Area and Period

**Figure 1:**
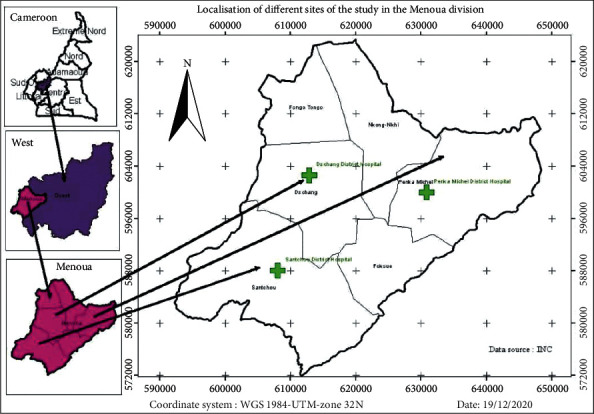
Sample collection location sites (A: Cameroon, B: West Region of Cameroon, and C: the six subdivisions of the Menoua division; turquoise circles represent study sites).

The Menoua division, one of the six divisions of the West Region of Cameroon, covers a surface area of 1380 km^2^; it is divided into six subdivisions (Figure 1) as follows: Dschang, Santchou, Nkong-Ni, Penka-Michel, Fokoue, and Fongo-Tongo. The altitude ranges from 600 to 800 m in Santchou through Dschang at 1500 m and Djuititsa at an altitude of 2200 m. The division has an average rainfall of about 1717.7 mm, and temperature ranges from 13.6°C to 25.35°C. About four in every five indigenes practice subsistence and/or smallholder farming, and the most important food stuffs grown include cabbage, carrot, onion, maize, banana, tomato, plantain, and beans. As of 2005, the division had a total population of 285,764. The capital of this division is Dschang.

This study was conducted from April 2018 to September 2019.

### 2.2. Study Design and Population

This was a hospital-based, cross-sectional study among febrile patients suspected of having typhoid fever. These patients had fever (temperature >37.5°C at inclusion) or reported febrile episodes in the past three days. Eligible patients who consented to study procedures were recruited and attributed a unique study code. This code was repeated instead of the patients' names or initials in all samples collected in the context of this study. The enrolment form was password secured and maintained by a study staff who was not associated with subsequent study procedures. A questionnaire containing demographic and clinical data and known risk factors was administered to each participant.

### 2.3. Assay Methods

#### 2.3.1. Blood Sample Collection

With the help of a sterile syringe and needle, about 2-3 ml of blood was collected from each participant as they arrived the laboratory. This was done by well-trained laboratory technicians independent of the study team.

#### 2.3.2. Test Procedure for the Widal Slide Agglutination Test

The qualitative slide agglutination test was performed using febrile patient antigen kits for *Salmonella typhi and Salmonella paratyphi* (TYDAL Widal Antigens for Slide and Tube tests, Spain). This test was used to determine the presence of anti-TO and TH antibodies in patients' sera. Collected blood was centrifuged using a bacteriological laboratory centrifuge (Andreas Hettich GmbH & Co. KG, 78532 Tuttlingen, Germany) at 5000 turns for 5 minutes. For the determination of *Salmonella* antibodies by slide agglutination, a drop of *Salmonella typhi* O and H antigens was added on a drop of serum on the slide card, rotated at 100 rpm for one minute, and recorded as either reactive or nonreactive.

#### 2.3.3. Test Procedure for the Typhidot (Immunoassay) Test (Typhidot^®^ Malaysian Biodiagnostic)

The Typhidot IgG and IgM rapid test device detects IgG and IgM antibodies to *S. typhi* and *S. paratyphi* through visual color development. Recombinant O and H antigens and antihuman IgG and antihuman IgM antibodies are used to detect the specific antibodies in human whole blood, serum, or plasma samples. The patient's serum sample was added to the sample well on the test panel, and specific IgG and/or IgM antibodies, if present, bind to the recombinant O and H antigens conjugated to colored particles on the sample pad. As the specimen migrates along the strip by capillary action and interacts with reagents on the membrane, the complex will be captured by antihuman IgG and/or antihuman IgM immobilized at the detection zone.

Venous blood was collected from patients suspected to have Salmonella infections (about 2-3 ml). The blood specimens were then centrifuged at 5000 rpm for 5 min using a laboratory centrifuge (Andreas Hettich GmbH & Co. KG, 78532 Tuttlingen, Germany). Using the provided pipette, one drop of serum was added to the test well on the test cassette followed by one drop of buffer. The setup was allowed for 15 minutes for migration across the membrane and color development in the result windows.

#### 2.3.4. Results' Interpretation

After 15 minutes, the test outcome was read as follows. A result was considered positive for IgG and IgM if one red band appeared on the control window (C) and two other red bands in both IgG and IgM windows, respectively. The shade of color may vary from pink to purple but indicates a positive result even with a faint line based on manufacturer's recommendations. IgM was considered to be positive when one red band appeared on the control window (C) and on the IgM window, while positive IgG was considered when one red band appeared on the control region (C) and on the IgG window. A negative result was indicated by the appearance of red color only on the control window. A test assay was considered invalid if the control line did not appear.

#### 2.3.5. Stool Sample Collection and Coproculture

Approximately 3 to 4 g of fresh stool was collected by febrile patients for the coproculture test after a brief training on how to collect the sample using a sterile wide-mouthed and transparent container. Assessors were provided to each of the participants. After collection, the stool was mixed in 3 ml normal saline, and 1 ml of this inoculum was diluted with 9 ml of freshly prepared Selenite F Broth (Oxoid CM0395B and LP0121A) onto prelabelled tubes and incubated at 37°C for 24 hours aerobically in a bacteriological incubator (Laboratory Incubator, DNP-9052-1, Midfield Equipment & Scientific, England) for amplification of bacterial growth. A loop full of inoculum from 3 ml normal saline was streaked on SSA agar (L: S-Biotech, USA) and MacConkey agar (Microxpress^®^, India) and incubated for 24 hours for the growth of colonies. After 24 hours, a loop full of bacterial culture from incubated tubes containing the selenite culture was restreaked into the *Salmonella-Shigella* agar (SSA) plates. The plates were examined carefully for the presence of characteristic colonies of *Proteus* spp., *E. coli, Salmonella* spp., *Citrobacter* spp.*, Shigella* spp.*, Klebsiella* spp., and *Serratia* spp. Suspected colonies were restreaked on *Salmonella-Shigella* agar for further identification and confirmation using API 20E gallery (bioMerieux, France). The technicians performing coproculture were blinded to the procedure and results of the Widal and Typhidot tests.

#### 2.3.6. Test Outcome Classification

The Widal test outcome was classified as positive or negative based on the slide reactivity of patient's serum. If, after the procedure, the patient's serum was reactive, it was noted as a positive test outcome. If the serum was nonreactive, it was noted as a negative test outcome. The procedure and classification were strictly followed for the total number of participants in the study.

The Typhidot immunoassay test outcome was determined by IgG and IgM positivity based on the manufacturer's guideline. Based on this guideline, positive IgM and/or IgM + IgG were considered definite diagnosis of typhoid fever. For the purpose of this study, positive IgG “only” was not considered positive for the reference test as it may reflect only past exposure and also because the test has no quantitative value.

The reference test was coproculture positive for *Salmonella typhi*/*paratyphi* and a history of fever (≥37.50 C) within 3–7 days or more including the hospital visit day. Other nontyphoidal *Salmonella*, although identified, did not constitute a positive test. They were therefore considered negative by the reference method. A positive *Salmonella typhi/paratyphi* coproculture and fever as determined by axillary temperature >37.5 C or reported fever within 3–7 days or more were considered the referent. We included these criteria to avoid spectrum bias as much as possible, given the spectrum of disease severity that may be present at the health facility and the likelihood of patients exposed to antibiotics before visiting the hospital.

#### 2.3.7. Statistical Analysis

We calculated the sample size based on the nomogram published by Carley et al., in 2003. Based on the nomogram for the sensitivity plot at alpha = 0.05, we estimated the sample size by using the following criteria: an estimated prevalence of typhoid fever of 15%, a confidence limit of 95%, and a type 1 error of 0.05. We obtained an estimated sample of 558 patients.

The primary outcome of this study was the test accuracy and the predictive values of the positive and the negative tests of the Widal slide agglutination test and the Typhidot immunoassay. Secondary outcomes were a comparison of the performance characteristics such as sensitivity, specificity, and likelihood ratios of positive and negative tests between the Widal and the Typhidot test in our setting. The area under the receiver operating curve was used to estimate the diagnostic accuracy of the Widal and Typhidot tests, while the sensitivity, specificity, and predictive values were calculated based on standard formulae. Regression analysis was used to assess risk factors of typhoid fever in the study population. All data were recorded in a predesigned questionnaire designed on Epi Info before transferring to GraphPad Prism 8.0.2 (GraphPad software 2019, California) for further processing. A *P* value <0.05 was considered significant for all comparisons.

## 3. Results

### 3.1. Sociodemographic Characteristics of Respondents

We enrolled a total of 558 patients presenting signs and symptoms suggestive of typhoid fever at the study sites (Table 1). The majority of the participants were female, accounting for 67.38% (376/558) of the total sample. The majority of participants were in the age range [11–20] years (219/558, 39.27%) and were students. According to occupation, respondents that were students were more represented (47.67% (*n* = 266)), followed by participants working in the private sectors (25.80% (*n* = 144)). Then, we had housewives accounting for 21.68% (*n* = 121) of the sample and finally civil servants representing only 4.83% (*n* = 27) of the total study population.

Based on the level of education, about half of our study participants had a secondary level of education (54.48%) followed by those who attained a tertiary level of education (31.18%) and then by those who had only a primary educational level (13.97%). The least represented were those who never went to school (0.17%). Married people were more represented (56.27%), followed by those who were single (37.27%), whereas widows were the least represented (6.45%) (Table 1).

### 3.2. Distribution of the Prevalence of Typhoid Fever in the Study Population

The distribution of patients according to gender shows a significant difference (*P* = 0.0148) between the male and female populations, with the male population being more infected (18.1%) compared to the female population (10.4%). Marital status also shows a significant difference (*P* = 0.0025), and the prevalence of typhoid fever among the unmarried population was higher (19.2%). Neither of the following factors: age group, occupation, or level of education were associated with typhoid fever (Table 2).

### 3.3. Clinical Signs and Symptoms of Study Participants

In this study, we recorded clinical signs and symptoms expressed by study participants. We estimated the frequency of these signs and symptoms, calculated the relative risk of acquiring typhoid fever given a symptom and presented the data in Table 3. As observed, febrile patients with abdominal pain had more than 2.5x the risk of typhoid fever compared to being febrile only. Other symptoms did not increased the risk of typhoid fever among the febrile patients. 

### 3.4. Performance of Widal and Typhidot Tests for Typhoid Fever Diagnosis

We found interesting comparisons between the Widal and Typhidot tests in their ability to diagnose typhoid relative to the reference test (Table 4). The overall sensitivity, specificity, and predictive values of the positive and negative tests were 80.56%, 94.03%, 66.6%, and 97.03%, respectively, for Typhidot-IgM and 94.44%, 48.35%, 21.32%, and 98.33%, for the Widal slide agglutination test. Cohen's kappa estimates were 0.386 (0.312–0.460) and 0.1660 (0.121–0.211) for the Typhidot immunoassay and Widal test, respectively. Of all observations, the percentage agreementsfor Typhidot immunoassay was 76.16% and 53.76% for the Widal test (Table 4).

### 3.5. Prevalence of Typhoid Fever in the Study Population

Out of the 558 participants in this study, 319 were positive for typhoid fever based on the Widal test with an antibody titer of 1 : 80 for both “O” and “H” antigens being taken as cutoff point values indicative of a recent typhoid infection giving a prevalence of 57.17%. On the contrary, 42.83% (*n* = 239) were negative for typhoid fever when Widal was used as the diagnostic tool. Interestingly, the prevalence of typhoid fever was 15.59% (n = 87) based on Typhidot-IgM and close to 12.90% (n = 72) based on the reference test. In Table 5, we summarise the differences in prevalence estimates by the two idea tests and the reference test.

As shown, the difference in estimating the prevalence of typhoid fever was significant (*P* = 0.0001) comparing the three diagnostic methods. The most likely estimate of thetrue prevalence estimate is that obtained with the reference method..

## 4. Discussion

Typhoid fever constitutes one of the major causes of morbidity and mortality in developing countries due to varied perceptions of the value of the Widal test in determining clinical decision-making. The clinical diagnosis of this disease remains difficult because its symptoms are similar to those of malaria and many other diseases [3, 21]. Laboratory confirmation of infection has relied on cheap slide agglutination tests which have their drawbacks leading to the appearance of false positive test results, the magnitude of which depends on the endemicity of the disease. Other tests such as the Typhidot immunoassay and the Tubex Widal dilution test do exist but have not been studied to understand their value in the diagnosis of typhoid fever in our setting. In the current study, we set out to compare the performance of the Widal slide agglutination test and the Typhidot test in patients suspected of typhoid fever in the Western Region of Cameroon.

Our study revealed disparities in the performance of the Widal slide agglutination test and the Typhidot test. Compared to the reference, the Widal test had a sensitivity of 0.9444 (0.8657–0.9782) and a specificity of 0.4835 (0.4394–0.5279), whereas the Typhidot assay had a sensitivity of 0.8056 (0.6997–0.8805) and a specificity of 0.9403 (0.9156–0.9581). Our results indicate that the Widal test performs poorly compared to the Typhidot test. Indeed, the result indicates that the Widal test correctly identifies about half of patients who are uninfected with the typhoid fever bacteria, while only about 6% of uninfected patients will be wrongly classified as having typhoid fever; meanwhile, they are uninfected. Also, the Widal test identifies 94% of febrile patients suspected of typhoid fever as positive, whereas only 80% of these patients were positive for the Typhidot immunoassay. However, only one in five febrile patients suspected for typhoid with a positive Widal test result truly had the disease (PPV = 21.3%), while two in every three febrile patients suspected for typhoid fever who have a positive Typhidot test truly had the disease (PPV = 66.7%). This low accuracy of the Widal test compared to the Typhidot test could result from exposure to cross-reacting antigens in febrile infections other than *Salmonella typhi*. Indeed, *Salmonella typhi* shares the O and H antigens with other nontyphoidal *Salmonella* and Enterobacteriaceae, as well as some parasites such as *Plasmodium falciparum* [22, 23], contributing to an increase in the probability of false positive results. Despite disparities in some parameters, we obtained similar estimates of the predictive values of the negative test for both Widal and Typhidot tests (98.3% and 97.0%, respectively). This indicates that both tests have the ability to rule out the disease in a population of suspected febrile patients.

The Widal test parameters obtained in the present study show some general patterns with some previous studies that are high sensitivity, low predictive value of the positive test, and high predictive value of the negative test. For example, in the study conducted by Muthaiyan [24] and collaborators, they reported a sensitivity of 88.5% (95% CI: (69.82–97.42)), a specificity of 81.56% (95% CI: (74.16–87.58)), a positive predictive value of 46.94% (95% CI: (32.54–61.720)), and a negative predictive value of 97.46% (95% CI: 92.74–99.44) for the Widal slide agglutination test. In a similar study in India in 2016 [24], the authors reported a sensitivity of 80.77% (95% CI: (60.64–97.42)), a specificity of 78.01% (95% CI: (70.22–84.54)), a positive predictive value of 40.38% (95% CI: (27.01–54.90)), and a negative predictive value of 95.65% (95% CI: ()90.14–98.56)). Sherwal et al. [25] also reported a sensitivity of 74%, a specificity of 83%, and a positive predictive value of 87.5%. Rahman et al. [26] obtained a sensitivity of 81.8%, a specificity of 69.2%, and a positive predictive value of 45.6%. This pattern generally shows that the Widal slide agglutination test, although highly sensitive, cannot be used to rule out the disease among those with positive results. Furthermore, the high predictive value of the negative test result indicates that this test can rule out the disease in patients that have a negative test outcome. Lastly, the test had a poor agreement indicated by the low Cohen's kappa value.

The prevalence has been shown to vary in different regions of Cameroon. For example, it was found to be 8.70% in Bamenda (northwest) [27], 21% in Buea, Southwest Region of Cameroon [28], and 2.5% obtained by Nsutebu et al. [29] in Tiko, Douala, and Yaoundé (Littoral and Centre Regions, respectively). Our estimate was also less than the prevalence obtained in a similar study in Nigeria [30]. Several reasons such as differences in sample size, risk factors, and environmental conditions could account for these disparities in prevalence. Although disparate, it is not likely that the pattern of performance parameters of both typhoid tests used in the present study may change from one region to another, although the magnitude of the performance parameters may be different. In the present study, five cases of confirmed typhoid fever by the reference test had negative Widal and Typhidot-IgM tests. These observations, although few in number, may result from early sampling before antibodies become detectable in blood as was reported in one study [31]. Furthermore, Andualem et al. found two cases of this category in a study comparing the Widal slide agglutination test against blood culture as the referent [32]. Although not frequent, this observation may also account for false negative results obtained by the index test in comparative studies of typhoid immunodiagnostics. Delay in antibody production in early disease may be the main determinant of this observation. Similar observations have been made recently in serology tests used to detect exposure to the COVID-19 virus [33].

Our study has some limitations. Firstly, standard blood culture has been recognised as the reference test for evaluating the diagnostic performance of typhoid fever tests. However, there are also problems associated with the test. It is first of all an invasive procedure [34] and has been demonstrated to have low sensitivity even when there is no exposure to antibiotics [19]. In addition, obtaining adequate amounts of blood from groups such as children is challenging. These make the use of such a test challenging in our setting. We used a composite reference of a positive stool culture and fever greater than or equal to 37.5°C or a history of fever in the past three to seven days. This is not the ideal reference either but represented the optimal combination under our local circumstances owing to its practicability. The search for a perfect composite reference for diagnostic comparisons is definitely still a way ahead even with emerging technologies [34]. Secondly, we did not have a way to check antibiotic exposure history data. We also did not collect clinical drug prescription data of these patients to quantify overtreatment and therefore the likely impact of treatment practices on our diagnostic accuracy estimations. This may likely have a small effect on our results which should be interpreted with caution.

## 5. Conclusion

We compared the diagnostic performance of the Widal slide agglutination test and Typhidot-IgM test against a coproculture, and the following results were obtained: the sensitivity of the Typhidot immunoassay was 80.56%, specificity was 94.03%, positive predictive value was 66.67%, and negative predictive value was 97.03%. The sensitivity of the Widal slide agglutination test was 94.44%, specificity was 48.35%, positive predictive value was 21.32%, and negative predictive value was 98.33%. Typhidot is a better alternative than the Widal test in the diagnosis of typhoid fever. The Widal test only rules out one of five typhoid fever suspected patients tested positive, as opposed to two of three who tested positive by the Typhidot immunoassay. Our results suggest that the Widal slide agglutination test, in its present configuration, does not have clinical benefits for typhoid fever confirmation in endemic regions such as Cameroon.

## Figures and Tables

**Table 1:** Sociodemographic characteristics of study participants.

Characteristics	Number (*n* = 558)	Frequency (%)
*Gender*		
Female	376	67.38
Male	182	32.61

*Age groups (years)*		
≤10	37	6.63
11–20	73	13.08
21–30	219	39.24
31–40	48	8.60
41–50	57	10.21
51–60	54	9.67
61–70	48	8.60
≥71	22	3.94

*Residence*		
Dschang	237	42.47
Fongo-Tongo	20	3.58
Nkong-Ni	76	13.62
Penka-Michel	12	2.15
Santchou	41	7.34

*Occupation*		
Student	266	47.67
Civil servant	27	4.83
Private sector	144	25.80
Housewife	121	21.68

*Level of education*		
None	1	0.17
Primary	78	13.97
Secondary	304	54.48
Higher education	174	31.18

*Marital status*		
Married	314	56.27
Single	208	37.27
Widow	36	6.45

**Table 2:** Prevalence of typhoid fever by sociodemographic characteristics in the study population.

	Total (*N* = 558)	*Salmonella typhi* positive (*n* = 72)	*P* value (chi^2^)
*Age groups (years)*			
<10	37	6 (16.2)	0.8010 (3.814)
[11–20]	73	7 (9.6)	
[21–30]	219	28 (12.8)	
[31–40]	48	5 (10.4)	
[41–50]	57	6 (10.5)	
[51–60]	54	8 (14.8)	
[61–70]	48	7 (14.6)	
>71	22	5 (22.7)	

*Gender*			
Female	376	39 (10.4)	0.0148
Male	182	33 (18.1)	

*Residence*			
Dschang	237	45 (19.0)	<0.0001 (29, 16)
Fongo-Tongo	20	1 (5.0)	
Nkong-Ni	76	10 (13.1)	
Penka-Michel	12	9 (75.0)	
Santchou	41	7 (17.1)	

*Occupation*			
Student	266	33 (12.4)	0.5105 (2.310)
Civil servant	27	6 (22.2)	
Private sector	144	17 (11.8)	
Housewife	121	16 (13.2)	

*Level of education*			
Not educated	1	0	0.9459 (0.3724)
Primary	78	11 (14.1)	
Secondary	304	40 (13.15)	
Higher education	174	21 (12.1)	

*Marital status*			
Married	314	28 (8.9)	0.0025 (11.95)
Single	208	40 (19.2)	
Widow/widower	36	4 (11.1)	

**Table 3:** Distribution of clinical symptoms of study participants.

	Total (*N* = 558)	Typhoid fever positive	*P* value	RR (95% CI)
Fever	558	72	0.9999	N/A
Headache	363	48	0.7927	1.074 (0.6854–1.699)
Nausea	107	12	0.633	0.843 (0.4691–1.475)
Abdominal pain	457	66	0.021	2.431 (1.130–5.401)
Fatigue	296	40	0.4444	0.827 (0.5365–1.279)
Vomiting	63	8	0.9999	0.9821 (0.4911–1.869)

*Type of stool*				
Hard	93	4	<0.0001
Mucoid	254	23	N/A	
Watery	170	44	<0.0001
Bloody	41	1		

*N*: total number of participants; RR: relative risk; N/A: not available.

**Table 4:** Performance of the Widal test and Typhidot immunoassay against the reference standard for the diagnosis of typhoid fever.

Stool culture		*P* value	Sen	Spec	PPV	NPV	LHR	Accu	Kappa (95% CI)	Agreement (%)
Pos	Neg									
*Widal*										
67	253	<0.0001	0.938 (0.87–0.98)	0.478 (0.44–0.53)	0.211 (0.17–0.26)	0.980 (0.96–0.99)	1.829	0.543	0.167 (0.12–0.21)	53.76
5	233									

*Typhidot-IgM*										
67	128	<0.0001	0.806 (0.70–0.88)	0.940 (0.92–0.96)	0.667 (0.56–0.76)	0.970 (0.95–0.98)	13.5	0.941	0.386	76.16
5	358								(0.31–0.46)	

PPV: positive predictive value; NPV: negative predictive value; Pos: positive; Neg: negative; LHR: likelihood ratio; Accu: accuracy; Sen: sensitivity; Spec: specificity; IgM: immunoglobulin M.

**Table 5:** Prevalence of typhoid fever based on the index and reference test methods.

Diagnostic test	Positive	Prevalence (%)	*P* value
Stool culture	72	12.90	<0.0001
Widal	319	57.17	
Typhidot-IgM	87	15.59	

## Data Availability

The datasets and/or analyzed data and materials used during the current study are available from the corresponding author upon reasonable request.

## Abbreviations

IgG: Immunoglobulin G
IgM: Immunoglobulin M
SSA: *Salmonella-Shigella* agar.
